# Diagnostic accuracy of VIKIA® Rota-Adeno and Premier™ Rotaclone® tests for the detection of rotavirus in Niger

**DOI:** 10.1186/s13104-017-2832-1

**Published:** 2017-10-23

**Authors:** Adamou Lagare, Aissatou Moumouni, Jérôme Kaplon, Céline Langendorf, Pierre Pothier, Rebecca F. Grais, Bassira Issaka, Anne-Laure Page

**Affiliations:** 1 Centre de Recherche Médicale et Sanitaire (CERMES), 634 Bld de la Nation, YN034-BP, 10887 Niamey, Niger; 2 Epicentre, Maradi, Niger; 3 Centre National de Référence virus des gastro-entérites, CHU Dijon Bourgogne, 21070 Dijon, France; 4 AgroSup Dijon, PAM UMR A 02.102, Université de Bourgogne Franche-Comté, Dijon, France; 5 Epicentre, 75011 Paris, France

**Keywords:** Rotavirus, Sensitivity, Specificity, VIKIA, Rotaclone, RT-PCR

## Abstract

**Objective:**

We conducted a parallel evaluation of the diagnostic accuracy of VIKIA® Rota-Adeno, a rapid diagnostic test (RDT) and Premier™ Rotaclone® an enzyme immunoassay (EIA) using reverse transcription polymerase chain reaction (RT-PCR) as the reference standard. The study was part of a rotavirus surveillance project in Niger.

**Results:**

The sensitivity of both tests was 80.7%. After exclusion of one indeterminate result by visual reading, the specificity of the Premier™ Rotaclone® was 100% by visual or optical density readings and that of VIKIA® Rota-Adeno test was 95.5%. Inter-reader agreement was excellent for both tests (kappa = 1). Our results showed almost similar performance of the EIA and RDT when compared to RT-PCR. Hence, the VIKIA® Rota-Adeno could be a good alternative for use in peripheral health centres where laboratory capacity is limited.

## Introduction

Rotavirus gastroenteritis represents an important public health concern, especially in low-income countries with an estimated 215,000 annual deaths among children under 5 years of age primarily in Sub-Saharan Africa [1]. The WHO recommends the use of enzyme immunoassays (EIA) carried out in hospital laboratory facilities for rotavirus diarrhea surveillance [2]. In areas with decentralized health systems, hospital surveillance might miss an important proportion of diarrhea cases, which are often managed at the level of primary health centers [3, 4]. Initial evaluations of rotavirus detection by EIA and RDT mainly used culture, electron microscopy or viral RNA electrophoresis as the gold standard with good sensitivity and specificity [5, 6]. Recent evaluations using highly sensitive reference methods as reverse transcription polymerase chain reaction (RT-PCR) have shown lower sensitivity of RDTs and EIAs for the detection of group A rotavirus in stool specimens [7, 8]. Few studies have evaluated RDT and EIA tests in parallel to compare their performance on the same samples and showed similar performance for both tests [8, 9]. While some studies reported a high level of false positive results with the widely used RDT VIKIA® Rota-Adeno (bioMérieux, Marcy l’Etoile, France) [8, 10], other studies showed good sensitivity and specificity [7, 11].

We conducted surveillance of rotavirus in hospitals and health centers in Niger from 2010 to 2012 using the RDT VIKIA Rota-Adeno assay, which was selected based on the good performance data published at the time, and the possibility to use the test in decentralized settings. Considering the concerns raised about this test afterwards, we subsequently performed a parallel evaluation of the diagnostic accuracy of VIKIA® Rota-Adeno RDT and one of the WHO recommended EIA, the Premier™ Rotaclone® (Meridian Bioscience Inc, Cincinnati, Ohio, USA), compared to RT-PCR as reference standard, using specimens collected in the surveillance project.

## Main text

### Methods

#### Study design

This study was an evaluation of frozen stool specimens from children < 5 years of age with watery diarrhea and moderate to severe dehydration collected and tested with the VIKIA® Rota-Adeno assay during rotavirus surveillance in Niger [3]. A random sub-sample of 734 specimens collected between July 2010 and May 2011 were tested at the French national reference laboratory for enteric viruses in Dijon, France, for enteric viruses with Seeplex® Diarrhea-V ACE assay (Seegene, Seoul, Korea). We randomly selected 140 rotavirus positive and 140 rotavirus negative specimens based on the reference method in order to estimate an expected sensitivity of 85% with an accuracy of ± 6% and an expected specificity of 90% with an accuracy of ± 5%. Unfortunately, 8 negative samples and 21 positive samples could not be retrieved. Thus, 119 positive and 132 negative samples by RT-PCR were included in the study. Samples for this analysis were similar to the overall study population in terms of demographic characteristics.

Selected samples were tested with VIKIA® Rota-Adeno and Premier™ Rotaclone® tests at CERMES, Niamey, Niger following the manufacturer’s recommendations. Two experienced laboratory technicians independently read the results of the RDT and EIA, with a third reading in case of discrepancy. They were all blind to the results of other tests and reference method.

#### Rapid diagnostic test (RDT)

VIKIA® Rota-Adeno is a rapid, qualitative, chromatographic immunoassay for the simultaneous detection of rotavirus and adenovirus. The test was performed according to the manufacturer’s recommendations and the results were read visually within 10 min and interpreted according to manufacturer’s recommendations, including reading as a positive result only lines that showed the expected color.

#### Enzyme immuno assay (EIA)

The Premier™ Rotaclone® kit is the only multi-well rotavirus EIA kit approved by the US Food and Drug Administration for in vitro diagnosis, it uses monoclonal antibodies raised against rotavirus structural protein VP6. It is suited for analyses of large numbers of samples and the results can be read after 1 h. The assay was performed according to manufacturer’s recommendations and results of the Premier™ Rotaclone® test were read both visually (VR) and using an optical density (OD) spectrophotometer (Emax microplate reader with Softmax®Pro5 S/N E13193; Molecular Devices; California) and interpreted according to manufacturer’s recommendations.

#### Reference method

The reference method for the detection of rotavirus was the Seeplex® Diarrhea-V ACE assay (Seegene, Seoul, Korea), which is a commercial multiplex PCR system for the detection of the following human diarrheal viruses: astrovirus, group A rotavirus, enteric adenovirus and norovirus.

Briefly, viral RNA was extracted from 20% faecal suspensions in phosphate-buffered saline (PBS) using the NucliSENS® EasyMAG™ platform (bioMérieux, Marcy l’Etoile, France) according to the manufacturer’s instructions. Viral RNA was reverse transcribed using the RevertAid™ First Strand cDNA Synthesis Kit (Thermo Fisher Scientific, Waltham, MA, USA) and multiplex PCR was performed with the Seeplex® Diarrhea-V ACE system (Seegene, Seoul, Korea) according to the manufacturer’s instructions.

#### Data management and analysis

Results of the index and reference tests were recorded in an Excel database (MS Corporation, Seattle, Washington, USA). Data analysis was conducted in Stata® 12.1 (College Station, Texas, USA). Sensitivity and specificity were estimated by comparing the results of the index test to those of the reference method using the *diagt* command, which displays summary statistics for diagnostic tests and provides exact binomial confidence intervals. Inter-reader reproducibility was assessed using Cohen’s kappa coefficient.

## Results

The results of the VIKIA® Rota-Adeno and Premier™ Rotaclone® on the selected samples compared to RT-PCR are shown in Table 1. The agreement between the two readers was excellent with kappa of 1 for both tests by visual reading. For the Premier™ Rotaclone®, one sample gave an indeterminate result by visual reading, which was negative by optical density reading. All other samples were concordant between visual reading and optical density reading (kappa = 0.99).

**Table 1:** Performance of Vikia^®^ Rota-Adeno and Premier™ Rotaclone ^®^ compared to RT-PCR on frozen samples

	RT-PCR		Test performance
	Positive	Negative	Total % sensitivity (95% CI) % specificity (95% CI)
Vikia Rota-Adeno			80.7 (72.4–87.3) 95.5 (90.4–98.3)
Positive	96	6	102
Negative	23	126	149
Premier Rotaclone			80.7 (72.4–87.3) 100 (97.2–100)
Visual reading			
Positive	96	0	96
Negative	23	131	154
Indeterminate	0	1	1
Optical density			
Positive	96	0	96
Negative	23	132	155
Total	119	132	251

In order to assess possible sample selection bias and freezing/thawing impact on sample rotavirus status, a comparison of the initial VIKIA® Rota-Adeno test in the field for the purposes of surveillance with RT-PCR results on all specimens tested and those selected for the evaluation are shown in Table 2. There was no significant difference between the sensitivity and specificity of the RDT when calculated using all specimens or only those selected for the evaluation, suggesting absence of sample selection bias

**Table 2:** Comparison of the initial VIKIA^®^ Rota-Adeno test and RT-PCR results on all specimens tested during the surveillance project and those selected for the evaluation

	RT-PCR			Performance	
	Positive	Negative	Total	% sensitivity (95% CI)	% specificity (95% CI)
Initial Vikia Rota-Adeno					
All specimens				82.6 (76.5–87.7)	90.8 (88.1–93.1)
Positive	157	50	207		
Negative	33	494	527		
Total	190	544	734		
Selected specimens				84.9 (77.2–90.8)	92.4 (86.5–96.3)
Positive	101	10	111		
Negative	18	122	140		
Total	119	132	251		

The performance of VIKIA® Rota-Adeno and Premier ™ Rotaclone® tests using RT-PCR as gold standard illustrated in Table 1 showed equal sensitivity of 80.7% for both assays and a respective specificity of 95.5 and 100% for the RDT and EIA tests.

## Discussion

The sensitivity of both assays was in accordance with the results of previous studies using RT-PCR as reference standard [7–9]. This reduced sensitivity compared to what has been reported in the manufacturer inserts for both assays could be due to the lack of detection of certain genotypes by EIA and RDT. However, the 23 false negative specimens by both EIA and RDT were genotyped and showed the most frequent genotypes in our study (10 G2P[4], 8 G12P[8], 2 G3P[6], 2 G3+G9P[6] and 1 G9P[8]) [3], suggesting no clear association between lack of detection and particular genotypes. Instead, it is most probably due to the high sensitivity of the reference standard [8, 9] and notably to the ability of RT-PCR to detect samples with low viral loads that cannot be detected by RDT and EIA assays [7, 12]. Unfortunately, the rotavirus viral load in samples was not assessed during this study to confirm this hypothesis. However, the relationship between viral load (assessed by Cq value) and EIA positivity has been well established by other groups [12, 13]. Low viral loads of rotavirus were also found in healthy controls, which led these authors to conclude that RT-PCR-positive EIA-negative samples do not reflect illness attributable to rotavirus and that EIA is a better suited method for laboratory diagnosis of rotavirus-associated diarrhea.

While specificity of Premier™ Rotaclone® was perfect in our study, the specificity of VIKIA® Rota-Adeno was lower, but still acceptable for surveillance purposes. The specificity of VIKIA® Rota-Adeno found in our study was much better than the specificity reported in an Australian study (54.3%) [8]. The low specificity found there might be due to a momentary low quality of the product since other evaluations either before [9, 14], or after, using tests produced after optimization of the manufacturing process [7], reported a good specificity for this test. In our study, all six VIKIA® Rota-Adeno false positive results showed a weak signal, compared to 15 of 96 (16%) weak positive results among positive samples by RT-PCR. As with other RDTs, although weak lines could represent false positive results, any (colored) line should be considered positive for optimal sensitivity of the rapid test. Strict respect of the time for results reading of RDT could help to overcome misreading, which is 10 min in the case of VIKIA® Rota-Adeno test.

Thus, considering the similar sensitivity of the VIKIA® Rota-Adeno and Premier™ Rotaclone® compared to RT-PCR in this study and the lower but acceptable specificity of the RDT, the rapid test could be a good alternative for use in peripheral health centres where laboratory capacity is limited.

## Limitations

The main limitation of this study was the use of thawed specimens. Frozen samples might slightly change the test performance, as illustrated by the fact that 6 of the 23 false negative specimens in this sub-study were initially positive by RDT in the surveillance activities, while one initially false-negative sample was found positive when testing frozen specimens. However, the correlation between the results of the VIKIA® Rota-Adeno test in the field on fresh samples and results on frozen samples was good (kappa = 0.88), similar to the correlation between field and central laboratory results for quality control during the surveillance study (kappa = 0.83), suggesting that testing on frozen samples did not strongly affect test results.

## Abbreviations

EIA: enzyme immuno assay
RDT: rapid diagnostic test
RT-PCR: reverse transcription polymerase chain reaction
WHO: World Health Organization
RNA: ribonucleic acid
cDNA: complementary deoxyribonucleic acid
VR: visual reading
OD: optical density
